# Analysis of the mediating effect between ehealth literacy and health self-management of undergraduate nursing students’ mental health literacy

**DOI:** 10.1186/s12912-024-01920-1

**Published:** 2024-04-23

**Authors:** Shuang Zhang, WeiWei Wang, Shan Wu, Hong Ye, LiXiao Dong, JingRu Wang, XiaoTong Ning, HuiXia Cui

**Affiliations:** 1 School of Nursing, JinZhou Medical University, Jinzhou, Liaoning Province China; 2 Medical College, Nanchang Institute of Technology, Nanchang, Jiangxi Province China; 3 The First Affiliated Hospital of Jinzhou Medical University, Jinzhou, Liaoning Province China; 4 School of Nursing, Wannan Medical College, Wuhu, Anhui Province China

**Keywords:** Anxiety, Health literacy, Mental health, Nursing education, Nursing students, Questionnaire design, Self-management

## Abstract

**Background:**

Good health self-management positively affects the health of healthcare providers and their ability to manage their patients’ health. This study explored the relationship between ehealth literacy, health self-management skills, and mental health literacy among undergraduate nursing students. Some studies have confirmed the correlation between e-health literacy and health self-management skills, while mental health literacy may be correlated with both, and this study aims to explore the relationship between the three.

**Methods:**

A descriptive cross-sectional survey was conducted at a medical university in northwestern China among 385 Chinese undergraduate nursing students. Participants completed the General Information Questionnaire, the Adult Health Self-Management Skills Rating Scale, the Mental Health Literacy Rating Scale, and the eHealth Literacy Scale, and provided valid responses. The IBM SPSS 27.0 statistical software was used for data entry and descriptive analysis, t-test, ANOVA, and Pearson correlation analysis. The IBM Amos 26.0 was used to construct the mediation effect model, and the Bootstrap method was employed to test mediating effects.

**Results:**

Mental health literacy, ehealth literacy, and health self-management skills of undergraduate nursing students were at a moderate to high level. Mental health literacy, ehealth literacy, and health self-management were positively correlated. Mental health literacy, particularly, played a partial mediating role of 31.1% ( 95% CI [0.307–1.418] ) between ehealth literacy and health self-management.

**Conclusions:**

Undergraduate nursing students’ mental health literacy partially mediates the link between eHealth literacy and health self-management skills. Schools should emphasize the development of nursing students’ e-health literacy and mental health literacy in order to improve their health self-management skills, which will not only bring about a better health outcome for the students, but will also benefit the health of the social population.

## Background

Health is a basic human right and an invaluable asset that can impact one’s personal, social, and economic life. The World Health Organization (WHO) defines health as “a state of complete physical, mental, and social well-being and not merely the absence of disease or infirmity” [1]. Individuals should assume responsibility for their health and ensure the body achieves a state of balance and coordination, as self-management is the most cost-effective way to stay healthy.

According to the WHO, more than 60% of a person’s life expectancy depends on self-care management [2]. The theory of health management first originated in the United States in the late 1920s [3]. Since then, Corbin and Strauss [4] have applied the term “self-management” to medical and health-related areas which have been universally adopted. Health self-management is recommended for patients with chronic diseases and is effective [5]. Nowadays, there is an increasing awareness of the need for health self-management for the sick, healthy, older adults and young persons. Previous research has shown that students who have better health self-management skills while in college have better health as adults [6]. Conversely, students with poor health self-management skills may develop health problems such as obesity, high blood glucose, high blood lipids and high blood pressure in adulthood [7, 8]. University students are especially encouraged to cultivate healthy self-management skills because they can keep their minds healthy, boost the immune system, and lower the effects of stress [9]. However, studies on health self-management among university students, especially nursing students, are limited, albeit published studies have shown a need to strengthen these skills among this group [10]. As the preparatory workforce for future clinical care, developing good health self-management skills in undergraduate nursing students will lead to good health outcomes for both students and professional experts in patient health coaching [6]. Good health self-management skills involves acquiring good mental health literacy by using skills developed through electronic health literacy.

Research shows that undergraduate nursing students are at high risk for mental health problems such as anxiety and depression [11]. Mental health literacy is considered an important self-competency to mitigate these risks and comprises the knowledge, attitudes, and behaviors that individuals develop in promoting and coping with their own and others’ mental health [12]. People with higher mental health literacy are more likely to be able to manage any mental health issues, tend to have better mental health, thus mental health literacy is vital in facing stress and avoiding psychological problems [13].

Nowadays, college students are exposed to a lifestyle that can endanger their physical and mental health. For instance, the ubiquitous use of smartphones and the Internet can be convenient and useful for study but can result in information overload, misinformation, and addiction [14]. Therefore, students must learn to decipher and effectively use these tools appropriately so they do not become mentally burdened. Electronic health literacy (eHL) is proposed to assist in this regard. In 2006, Norman and Skinner [15] defined eHL as “the ability to search for, understand, and evaluate health information on electronic resources and to use the information obtained to address and solve health problems”. Studies described the benefits of eHL for healthcare providers and their patients and emphasized that students with this competency do better in later clinical work [16]. Therefore, developing and improving eHL in undergraduate nursing students is an important task for nursing educators.

**Fig. 1:**
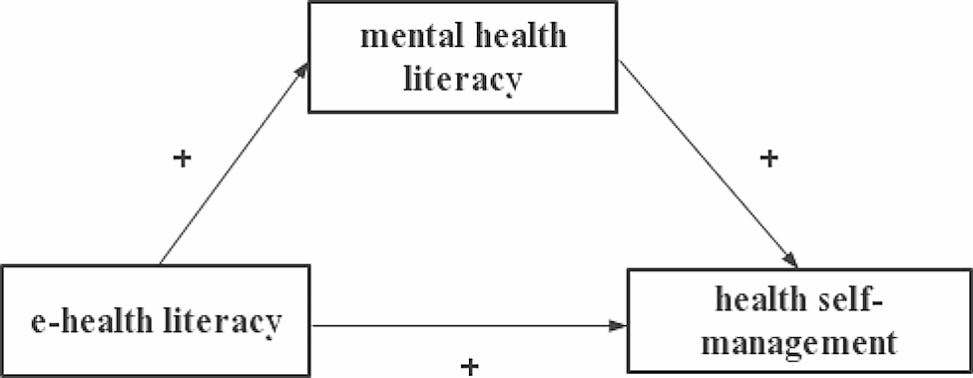
Moderation model and hypotheses

Several studies have shown positive correlations between eHL and mental health literacy [17], between eHL and health self-management skills [18], and between mental health literacy and health self-management skills [19]. However none of the study populations were undergraduate nursing students. As the mainstay of future nursing professionals, nursing students should possess good health self-management skills. These are beneficial to their health status and allow them to better educate and guide future patients and improve their health outcomes. Based on the previous arguments, we hypothesized that mental health literacy mediates the relationship between eHL and health self-management skills (see Fig. 1). Moreover mental health literacy plays a mediating effect between eHL and health self-management skills. Thus, if mental health literacy plays an important role in mediating the relationship between eHL and health self-management competencies, then we might consider interventions such as mental health literacy to improve health self-management competencies among undergraduate nursing students.

## Methods

### Study design and participants

This study employed a descriptive cross-sectional research design. Undergraduate nursing students (*N* = 407) were recruited via convenience sampling from a medical university in northeastern China. Only full-time registered students who were at least 18 years and willing to participate were included. Following the Kendall sample estimation principle [20], the sample size was at least 10 to 20 times the number of variables, plus 20% for sample attrition. The calculated value range was 204 to 408; 385 valid responses were collected. Participants were between 18 and 26 years old, with a mean age of (21.28 ± 1.23) years; 31(8.1%) were male and 354(91.9%) were female.

### Ethical considerations

The study was performed in accordance with the Declaration of Helsinki and was approved by JinZhou Medical university ethics committee. The study followed the principles of voluntariness and involved minimal risk. The questionnaire was anonymized to protect participant privacy. All the participants provided informed consent. All methods were carried out in accordance with relevant guidelines and regulations.

### Data collection

**Fig. 2:**
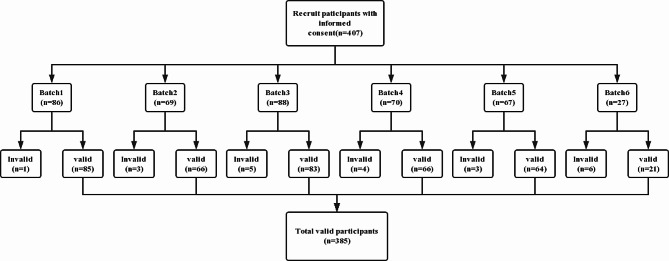
Participant recruitment flow chart

The researcher obtained the course schedules of undergraduate nursing students in advance with the help of the course instructors. They also helped to organize the students by establishing batches in the classrooms and assisting with filling out the questionnaires. Data gathering occurred from October to December 2021 when the students were on break. Questionnaires were distributed, completed, and collected on-site. Six batches were organized [ Batch 1 = 86; Batch 2 = 69; Batch 3 = 88; Batch 4 = 70; Batch 5 = 67; Batch 6 = 27 ]; 22 students were excluded due to irregular completion or omission of content (see Fig. 2). The questionnaire efficiency rate of the responses was 94.6%.

### Data collection instruments

The researcher designed the general information questionnaire. The questionnaire requested socio-demographic details such as age, gender, height, weight, grade, household location, and social parameters such as students’ role in their family (e.g., first/second child), positions in school (e.g., student leaders), study pressure, and personal habits (e.g., insomnia).

The Adults Health Self-Management Skill Rating Scale (AHSMSRS), developed by Chinese scholar QiuLi Zhao et al. [21] in 2011 was used to assess health self-management competence. This consisted of three subscales and seven dimensions (38 items). The three subscales consisted of health self-management behaviors: three dimensions of diet self-management, exercise self-management, and disease coping (14 items); health self-management environment - two dimensions of resource use and environmental self-management (10 items), and health self-management perceptions - two dimensions of health beliefs and self-efficacy (14 items). The scale was based on a 5-point Likert scale with a score range of 38 to 190, with higher scores representing greater health self-management ability in adults. Cronbach’s coefficient for this scale was 0.950.

Mental health literacy was assessed using an adolescent mental health literacy assessment scale developed by Chinese scholar Danlin Li [22]. This was based on a conceptual framework of mental health literacy and a theoretical model of knowing, believing, and acting, based on an extensive literature review, group discussions, and expert interviews. The scale included 22 items in four dimensions: knowledge, identification, attitude, and behavior, and was scored on a 5-point Likert scale with a score range of 22 to 110, with higher scores indicating higher levels of mental health literacy. The scale was consistent with local Chinese cultural habits and had good reliability. It is widely used among Chinese high school and college students [22, 23]. The Cronbach’s coefficient was 0.847.

Norman and Skinner [15] developed the first self-assessment tool for measuring eHL. In this study, it was used to assess eHL. The eHealth Literacy Scale has been translated into at least seven languages and is widely used. In 2013, Chinese scholars Shuaijun Guo et al. developed a Chinese version of the scale to form three dimensions: the ability to apply online health information and services, the ability to judge, and the ability to make decisions (8 items) [24]. The entries were scored on a 5-point Likert scale with a score range of 8 to 40, with higher scores indicating better eHL. The Cronbach’s coefficient was 0.955.

### Statistical methods

The SPSS 27.0 (IBM) statistical software was used for data analysis. The data were tested for normality by plotting histograms. Count data were described by frequency and composition ratios. The measures conformed to a normal distribution and were described as mean ± standard deviation; t-test and ANOVA were used for one-way analysis. Pearson correlation was used for correlation analysis, and the test level α = 0.05.

IBM Amos 26.0 was applied to construct the mediating effect model and the Bootstrap method was used to test the mediating effect. Relative chi-square (CMIN/DF), root mean squared error of approximation (RMSEA), goodness of fit index (GFI), adjusted goodness of fit index (AGFI), incremental fit index (IFI), Tucker-Lewis-index (TLI) and comparative-fit-index (CFI) were used for model fit evaluation.

## Results

### Participant information

There were statistically significant differences in health self-management skills assessment scores among the 385 undergraduate nursing students in different grades, study pressure, and late-night situations (*P* < 0.05). There were no statistically significant differences in health self-management skills scores on the remaining factors (*P* > 0.05) (see Table 1).

**Table 1:** General information of undergraduate nursing students (*n* = 385)

Characteristics	Categories	N(%)	M ± SD	F/t	*P*
Gender	Male	31(8.1)	147.61 ± 3.93	0.622^a^	0.535
	Female	354(91.9)	144.98 ± 22.67		
BMI	< 18.5	75(19.5)	143.23 ± 24.84	0.718^b^	0.489
	18.5∼	224(58.2)	146.35 ± 21.13		
	≥ 24	86(22.3)	143.90 ± 24.27		
Grade(years)	Freshman year	234(60.8)	147.24 ± 22.42	4.860^b^	**0.002**
	Sophomore	66(17.1)	145.36 ± 22.76		
	Junior	64(16.6)	135.80 ± 22.55		
	Senior Year	21(5.5)	150.52 ± 17.29		
Account Location	Cities and towns	174(45.2)	147.10 ± 22.25	1.510^a^	0.132
	Rural	211(54.8)	143.62 ± 22.81		
Only child	Yes	181(47.0)	146.72 ± 21.62	1.254^a^	0.211
	No	204(53.0)	143.83 ± 23.39		
Student leaders	Yes	174(45.2)	144.79 ± 23.36	0.099^a^	0.753
	No	211(54.8)	145.52 ± 21.99		
Study pressure	Heavy	40(10.4)	136.85 ± 27.07	4.193^b^	**0.016**
	General	292(75.8)	145.40 ± 20.92		
	None	53(13.8)	150.36 ± 26.25		
Stay up late	Yes	134(34.8)	136.10 ± 23.43	−5.800^a^	**< 0.001**
	No	251(65.2)	150.05 ± 20.59		

Note: ^a^*t*-value,^b^*F*-value

### Scores for each scale

The mean score of the total entries of the AHSMSR Scale was (3.82 ± 0.59). The behavioral subscales scored the lowest among the subscales, with the lowest score being the exercise management dimension, followed by the diet management dimension (see Table 2).

**Table 2:** Health self-management competency scale scores (M ± SD)

	Minimum	Maximum	Score	Average score of items
Health self-management	78	190	145.19 ± 22.59	3.82 ± 0.59
Cognitive subscale	26	70	57.53 ± 9.39	4.11 ± 0.67
Self-efficacy	5	25	20.83 ± 3.72	4.17 ± 0.74
Health Beliefs	18	45	36.70 ± 6.22	4.08 ± 0.69
Environment subscale	18	50	39.38 ± 7.19	3.94 ± 0.72
Environmental Management	10	25	20.48 ± 3.59	4.09 ± 0.72
Resource Utilization	5	25	18.90 ± 4.25	3.78 ± 0.85
Behavior Subscale	22	70	48.28 ± 9.81	3.45 ± 0.70
Disease coping	4	20	16.20 ± 3.10	4.05 ± 0.77
Diet management	5	25	17.44 ± 4.25	3.48 ± 0.85
Exercise management	5	25	14.64 ± 5.09	2.92 ± 1.02

The mean score for the entries on mental health literacy was (3.80 ± 0.52), with lower scores for the identification and attitude dimensions (see Table 3).

**Table 3:** Mental health literacy scale scores (M ± SD)

	Minimum	Maximum	Score	Average score of items
Mental health literacy	49	110	83.52 ± 11.34	3.80 ± 0.52
Behavioral dimension	5	25	19.54 ± 3.15	3.91 ± 0.63
Knowledge dimension	6	30	23.31 ± 3.82	3.89 ± 0.64
Identity dimension	5	25	18.64 ± 3.52	3.72 ± 0.70
Attitude dimension	6	30	22.03 ± 6.11	3.67 ± 1.02

The mean score for the total entries of eHL was (3.82 ± 0.76), with the lowest score for the decision-making dimension (see Table 4).

**Table 4:** eHealth literacy scale scores (M ± SD)

	Minimum	Maximum	Score	Average score of items
E-Health literacy	8	40	30.59 ± 6.12	3.82 ± 0.76
Application	5	25	19.22 ± 3.96	3.84 ± 0.79
Judgment	2	10	7.68 ± 1.64	3.83 ± 0.82
Decision	1	5	3.69 ± 0.89	3.69 ± 0.89

### Correlation analysis of eHealth literacy, mental health literacy, and health self-management skill

There was a positive correlation between the eHL, mental health literacy, and health self-management skill total scales and its three subscale scores among undergraduate nursing students (*P* < 0.01)(see Table 5).

**Table 5:** Pearson correlation analysis between variables

	HSM	HSM−1	HSM−2	HSM−3	EHL	MHL
HSM	1.000					
HSM−1	0.816**	1.000				
HSM−2	0.892**	0.578**	1.000			
HSM−3	0.870**	0.478**	0.778**	1.000		
EHL	0.236**	0.110*	0.243**	0.269**	1.000	
MHL	0.344**	0.210**	0.309**	0.372**	0.248**	1.000

Note: **Significant at *P* < 0.01, *Significant at *P* < 0.05

HSM: Health self-management, HSM−1: Health self-management behaviors subscale, HSM−2: Health self-management environment Subscale, HSM−3: Health self-management perceptions subscale, EHL: EHealth literacy, MHL: Mental health literacy

### Analysis of the mediating effect of mental health literacy in the relationship between eHealth literacy and health self-management

The results of the model fitness test showed that the mediated effects model fit well (see Table 6).

**Table 6:** Model fitness test

Indicators	Reference values	Measured values	Judgment
CMIN/DF	< 3.000	1.810	yes
RMSEA	< 0.080	0.046	yes
GFI	> 0.900	0.970	yes
AGFI	> 0.900	0.948	yes
IFI	> 0.900	0.986	yes
TLI	> 0.900	0.980	yes
CFI	> 0.900	0.986	yes

The path coefficients between eHL and mental health literacy, mental health literacy and health self-management, as well as eHL and health self-management, were evenly statistically significant (*p* < 0.01). Mental health literacy had a partially mediating effect between eHL and health self-management (see Table 7).

**Table 7:** Structural equation model path relationship test results

Path relationship test	Standardized Estimate	SE	CR	*P*
MHL<---EHL	0.247	0.258	4.305	< 0.01
HSM<---MHL	0.362	0.116	5.653	< 0.01
HSM<---EHL	0.198	0.448	3.603	< 0.01

**Fig. 3:**
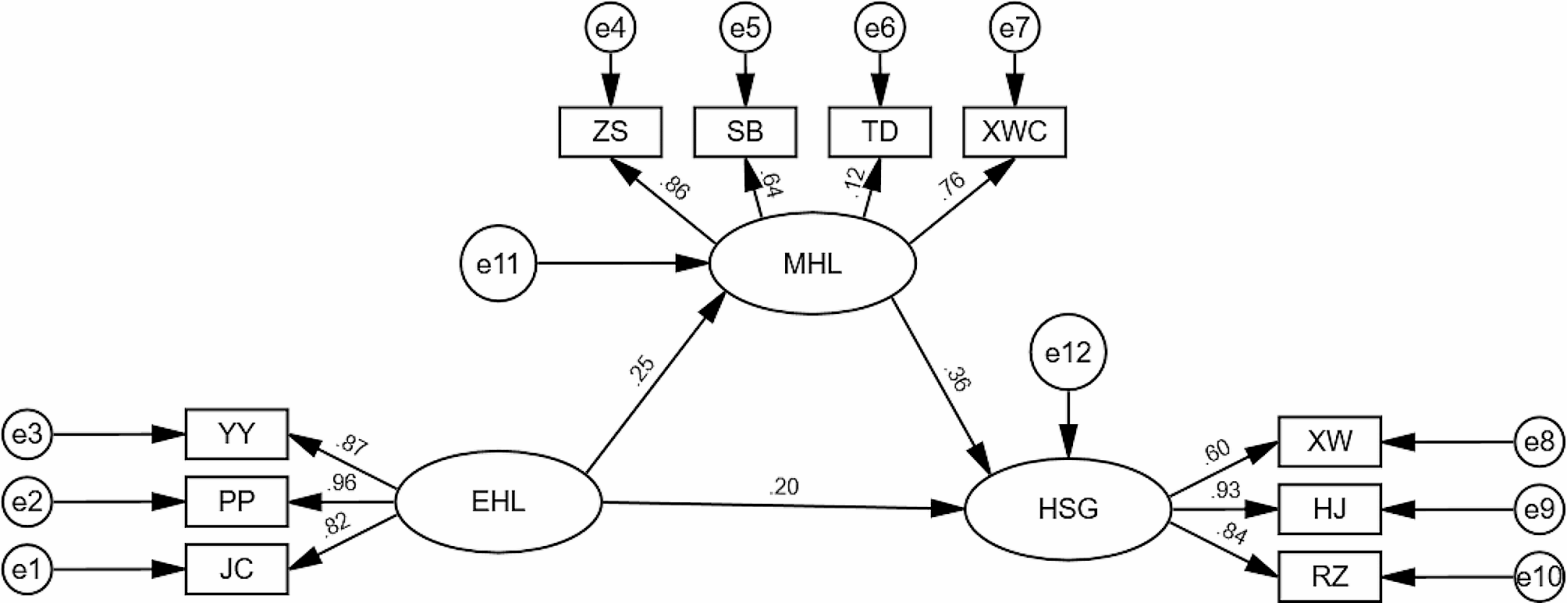
Diagram of the mediating effect model. Note: HSM: health self-management; MHL: mental health literacy; EHL: E-health literacy

The results of the Bootstrap mediating effect test showed that the direct effect accounted for 68.9% of the total effect and the indirect effect accounted for 31.1% (see Table 8). The mediating effect plot is shown in Fig. 3.

**Table 8:** Results of Bootstrap mediated effects test

Parameter	Estimate	Lower	Upper	*P*	Effect ratio(%)
Indirect effect	0.727	0.307	1.418	0.001	31.1
Direct effect	1.613	0.639	3.002	0.002	68.9
Total effect	2.339	1.258	3.776	0.001	100.0

## Discussion

The findings showed the health self-management skills of these 385 Chinese undergraduate nursing students were at an intermediate to a high level. There was a higher score than one for health self-management in patients with hypertension [25], and also better self-management in patients with diabetes [26]. This suggests that undergraduate nursing students have better health self-management skills, which may be attributed to their professional studies. However, the health self-management skills of the nursing undergraduates were poorer than those of the healthcare undergraduates [10], and this infers a need to strengthen this trait.

EHL was positively correlated with health self-management skills, which is consistent with the research by Scholar Wu [27]. This also aligns with the findings of Li scholars that people with higher eHL had stronger health self-management skills [28]. This may be because of their ability to make full use of e-health resources, to extract and process the information they need from a multitude of information platforms and resources [18]. Accordingly, they are better equipped to strengthen their physical health conditions and adopt healthy behaviors, thus improving their health self-management.

EHL was positively correlated with mental health literacy, which follows Alijanzadeh’s findings [29]. This is also compatible with the findings of Balay that undergraduate nursing students with higher eHL had higher levels of mental health literacy [30]. The analysis showed students with high e-health literacy tend to have access to more health knowledge. This forms the basis of psychology, and with the support of health knowledge, students are better able to accurately analyze and rationally deal with psychological stress or mental stimuli.

Mental health literacy was positively correlated with health self-management skills, which is aligned with scholar Xie’s study [31]. Chu showcased similar findings in their research that showed young people with higher levels of mental health literacy had stronger health self-management skills [32]. Physical and mental health are closely related. The higher the level of mental health literacy the better the mental health of students and the more actively they manage their health.

In summary, by examining the partial mediating role of mental health literacy, this study was able to better explain the role relationship between eHL and health self-management skills. EHL can influence health self-management through mental health literacy, in addition to directly influencing health self-management among undergraduate nursing students. Health self-management skills of undergraduate nursing students can be better enhanced by intervening in the mediating variable of mental health literacy. Consequently, nursing educators can provide undergraduate nursing students with books, training and counseling when necessary that are conducive to improving their mental health literacy, etc. This study adds to the paucity of literature on self-management techniques for nursing students and provides further information to improve their health self-management skills.

### Limitations

Due to time and manpower constraints, this study only facilitated a sample of students from a medical university in northeastern China. Consequently, the study population and the sample selection were relatively limited. Future studies could entail multi-center, large-sample surveys. Second, since this was a cross-sectional study, the survey was conducted at one point in time and was unable to show a causal relationship. In the future, a longitudinal study could explore the causal relationship between the variables.

### Strengths

The increasing number of global health workers who are also faced with chronic health conditions is of concern to diverse stakeholders. Accordingly, our research attempted to investigate the level of eHealth Literacy, Mental Health Literacy, and health self-management skills among a sample of undergraduate student nurses in China. We felt this was an important demographic given this group represents the future workforce, and so their level of knowledge was important, not only for self-management but also in their treatment of future patients. Then, while we understand health may be perceived as a personal decision, we felt the educational institutions had some culpability to encourage healthier choices and behaviors among this demographic. Accordingly, we felt the research would raise awareness among educators about how these sociodemographic factors affect students’ literacies.

## Conclusions

This study reports on the mediating effect of mental health literacy between eHL and health self-management skills among undergraduate nursing students. Nursing educators can not only improve undergraduate nursing students’ health self-management skills by improving their e-health literacy, but they can also better improve undergraduate nursing students’ health self-management skills by intervening in their mental health literacy.

## Data Availability

The datasets used and/or analysed during the current study are available from the corresponding author on reasonable request.

## Abbreviations

WHO: World Health Organization
eHL: eHealth literacy
AHSMSRS: Adults Health Self-Management Skill Rating Scale
HSM: Health self-management
HSM-1: Health self-management behaviors subscale
HSM-2: Health self-management environment Subscale
HSM-3: Health self-management perceptions subscale
MHL: Mental health literacy
